# High-Field High-Repetition-Rate Sources for the Coherent THz Control of Matter

**DOI:** 10.1038/srep22256

**Published:** 2016-02-29

**Authors:** B. Green, S. Kovalev, V. Asgekar, G. Geloni, U. Lehnert, T. Golz, M. Kuntzsch, C. Bauer, J. Hauser, J. Voigtlaender, B. Wustmann, I. Koesterke, M. Schwarz, M. Freitag, A. Arnold, J. Teichert, M. Justus, W. Seidel, C. Ilgner, N. Awari, D. Nicoletti, S. Kaiser, Y. Laplace, S. Rajasekaran, L. Zhang, S. Winnerl, H. Schneider, G. Schay, I. Lorincz, A. A. Rauscher, I. Radu, S. Mährlein, T. H. Kim, J. S. Lee, T. Kampfrath, S. Wall, J. Heberle, A. Malnasi-Csizmadia, A. Steiger, A. S. Müller, M. Helm, U. Schramm, T. Cowan, P. Michel, A. Cavalleri, A. S. Fisher, N. Stojanovic, M. Gensch

**Affiliations:** 1 Helmholtz-Zentrum Dresden-Rossendorf, Bautzner Landstr. 400, 01328 Dresden, Germany; 2 Deutsches Elektronen Synchrotron, Notkestr. 85, 22607 Hamburg, Germany; 3 European XFEL GmbH, Albert-Einstein-Ring 19, 22761 Hamburg, Germany; 4 Freie Universität Berlin, Arnimallee 14, 14195 Berlin, Germany; 5 Karlsruhe Institute of Technology, Kaiser Str. 12, 76131 Karlsruhe, Germany; 6 Max Planck Institute for the Structure and Dynamics of Matter, Luruper Chaussee 149, 22761 Hamburg, Germany; 7 Eotvos Lorand University, Pazmany s 1/C. 1117 Budapest, Hungary; 8 Helmholtz-Zentrum Berlin, Albert-Einstein Str. 15, 12489 Berlin, Germany; 9 Fritz-Haber-Institut der Max-Planck-Gesellschaft, Faradayweg 4–6, 14195 Berlin, Germany; 10 Gwangju Institute of Science and Technology (GIST), Gwangju 500–712, Korea; 11 ICFO–Institute of Photonic Sciences, Av. Carl Friedrich Gauss 3, 08860 Castelldefels (Barcelona), Spain; 12 Physikalisch-Technische Bundesanstalt (PTB), Abbestr 2–12, 10587 Berlin, Germany; 13 SLAC National Accelerator Laboratory, 2575 Sand Hill Rd, Menlo Park, California 94025, USA; 14 S. P. Pune University, Pune 411 007, India; 15 Technische Universität Dresden, 01062 Dresden, Germany; 16 University of Groningen, 9747 AG Groningen, Netherlands; 17 Max Planck Institut für Festkörperforschung, Heisenbergstr. 1, 70569 Stuttgart, Germany; 18 Universität Stuttgart, Pfaffenwaldring 57, 70550 Stuttgart, Germany

## Abstract

Ultrashort flashes of THz light with low photon energies of a few meV, but strong electric or magnetic field transients have recently been employed to prepare various fascinating nonequilibrium states in matter. Here we present a new class of sources based on superradiant enhancement of radiation from relativistic electron bunches in a compact electron accelerator that we believe will revolutionize experiments in this field. Our prototype source generates high-field THz pulses at unprecedented quasi-continuous-wave repetition rates up to the MHz regime. We demonstrate parameters that exceed state-of-the-art laser-based sources by more than 2 orders of magnitude. The peak fields and the repetition rates are highly scalable and once fully operational this type of sources will routinely provide 1 MV/cm electric fields and 0.3 T magnetic fields at repetition rates of few 100 kHz. We benchmark the unique properties by performing a resonant coherent THz control experiment with few 10 fs resolution.

The advent of laser-based coherent THz emission and THz detection schemes more than 25 years ago^1^,^2^ has revolutionized linear spectroscopy and imaging^3^ in the THz frequency range and helped to improve our fundamental understanding of matter tremendously. THz pulses have since been utilized as gentle sensitive probes of fundamental low-frequency excitations in solids, liquids or gases^4^,^5^,^6^,^7^. Key of the success of this technology is the possibility to generate and detect these THz pulses coherently, thereby providing an excellent dynamic range and circumventing detection of the large intrinsic thermal background in the THz regime.

More recently, the generation of THz pulses with much higher field amplitudes has become possible, which permitted a conceptually new approach for investigating low energy degrees of freedom in matter^8^,^9^. In this approach, the high THz-fields are utilized for the driving (rather than probing) of these modes to much higher amplitudes so that e.g. the induced coherent dynamics of a particular low energy degree of freedom can be visualized directly in the time domain. Thereby fascinating examples of so called coherent THz control could be demonstrated (e.g. coherent THz control of spins^10^). One particular class of experiments aims to utilize the much higher THz field to drive the low energy modes into the nonlinear regime which has been shown to allow for the selective control of macroscopic fundamental material properties^11^. Recent work covers a broad range of topics from controlling transient macroscopic electronic or magnetic phases in solids^12^,^13^,^14^,^15^,^16^,^17^ to influencing biological function in proteins^18^. The universal concept in such control experiments is that the excitation of matter with ultrashort and strong electric and magnetic fields but low photon energies, may either act as a quasi-DC field or as a mode-selective driving force of collective low-energy degrees of freedom into an extreme nonequilibrium. When combined with appropriate probes for the induced transient states, for example photoelectron spectroscopy in the case of complex solids^19^,^20^,^21^, fundamental mechanisms such as high temperature (HT_C_) superconductivity may thereby be elucidated by separating entangled degrees of freedom by their characteristic time- and energy scales^22^,^23^.

Presently, the most commonly employed high-field THz sources are based on nonlinear-optical conversion of near-infrared (NIR) femtosecond laser pulses. These schemes produce carrier envelope phase (CEP) stable single cycle and multi-cycle THz pulses at high pulse energies/field strength. However, the relatively low repetition rates of typically 1 kHz (refs 8,9 and references therein) limit the sensitivity of ultrafast experiments to those phenomena exhibiting rather drastic changes in the transient linear optical properties of the samples. Providing high THz fields at elevated repetition rates would allow one to observe more subtle changes in the ultrafast response^8^,^24^. The high duty cycle would enable use of more specific ultrafast probes such as time-resolved Raman scattering^25^, time-resolved near-field microscopy^26^, time-resolved photoelectron spectroscopy^19^,^27^,^28^ or time-resolved infrared difference spectroscopy^29^,^30^, just to name a few.

Enabling higher repetition rates for the THz pump pulse and thereby allowing for a more thorough and precise spectroscopic characterization of the induced exotic transient states is to this end a crucial development (see e.g.^23^). In this paper we show that compact modern electron accelerators allow simultaneous operation of multiple radiators for high-field THz pulses at repetition rates up to the MHz regime. The source parameters exceed those of existing techniques by several orders of magnitude as shown in Fig. 1b. The working principle is well suited to operation as multi-user facility for communities working in the field of THz driven dynamics. By providing CEP-stable, high-field THz pulses with femtosecond timing to non-laser experts, this new class of sources will eventually lead to a considerable widening of the application of transient THz fields in the material and life sciences, analogous to the role synchrotron storage rings have played in establishing spectroscopic techniques based on VUV and X-ray radiation.

## Results

The current prototype facility aims for the generation of strong THz field transients with two different THz sources operating in parallel. The unique combination of high-field amplitude and high-repetition rate is achieved by complementing superconducting radiofrequency (SRF) accelerator technology (providing cw operation with repetition rates in the MHz regime) with superradiant THz sources^31^ (which have shown to provide THz pulse energies in the few 100 μJ or even mJ regime^32^,^33^ but at repetition rates of few 10 Hz).

**Figure 1:**
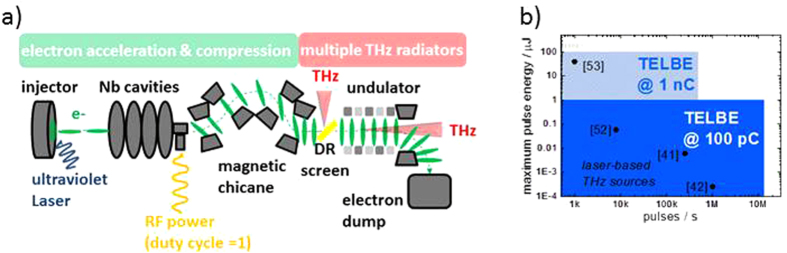
Schematic of a compact high-repetition-rate accelerator-driven THz source and comparison of pulse energies with record pulse energies of laser-driven sources. (**a**) Electron bunches are extracted from a solid, accelerated to relativistic energies and compressed to sub-ps duration in a compact SRF linac with a chicane bunch compressor. The electron bunches can emit THz pulses in different types of radiators. At TELBE, repetition rates up to 13 MHz are feasible. THz pulses are generated by a diffraction radiator (DR) and one undulator. (**b**) Comparison between laser-based sources (black dots) and TELBE. Laser-based sources operating higher than 10 kHz repetition rate are limited to pulse energies <10 nJ, at repetition rates above 250 kHz to <0.25 nJ. TELBE currently exceeds these values by more than 2 orders of magnitude (blue-shaded). The high- charge-mode-of-operation will enable pulse energies of 100 μJ (light-blue-shaded).

**Figure 2:**
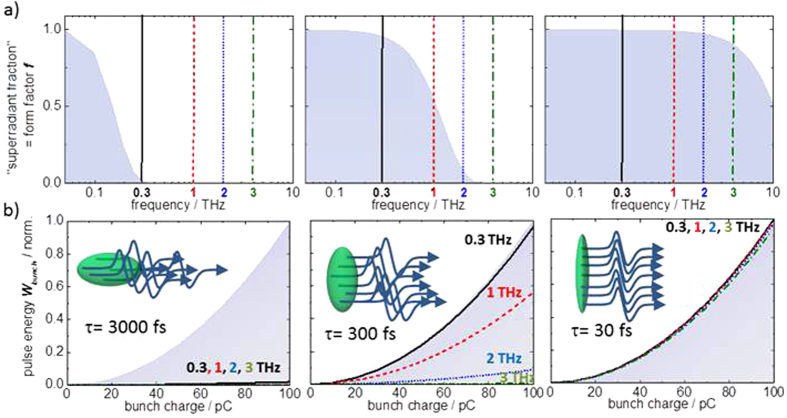
Fundamental principle of superradiance. Superradiant emission from electron bunches becomes significant for frequencies sufficiently lower than the inverse of the bunch duration τ. Following equation (1) the emission scales quadratically with the charge at low enough frequencies but diminishes at higher frequencies when a smaller fraction of the charge fits within the wavelength. This behavior can be described by the dimensionless form factor ***f***. (**a**) Form factors plotted for an assumed Gaussian bunch form with duration (FWHM) of 3000 fs, 300 fs and 30 fs (grey-shaded). (**b**) Corresponding dependence of the pulse energies at THz frequencies of 0.3 THz (black solid), 1 THz (red-dashed), 2 THz (blue-dotted) and 3 THz (green-dash-dot) on the bunch charge. For simplicity a “white” radiator with a frequency independent emission characteristic is assumed. The upper edge of the blue-shaded area corresponds to the case of a form factor equal to 1.

A schematic of a high-repetition-rate superradiant THz source is given in Fig. 1a. Electron bunches are generated, chirped and accelerated to relativistic energies in SRF cavities made from Niobium (Nb) and finally compressed in one or several magnetic chicanes to sub-ps duration. The compact SRF accelerator is followed by multiple THz radiators. The repetition rate and bunch charge can be freely adjusted in SRF accelerators according to the requirements of the experiment up to maximum values limited by the employed electron injector. At the prototype facility, the two available injector types permit operation up to 13 MHz at 100 picocoulomb (pC) charge (thermionic injector^34^) and soon 500 kHz at up to 1 nanocoulomb (nC) charge (SRF injector^35^). The electrons can be accelerated to a maximum energy of 40 MeV. The emitted pulse energy in a bunch scales according to the following law:


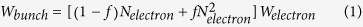


where ***N***_***electron***_ is the number of electrons and ***W***_***electron***_is the emission characteristics of the radiator for a single electron (31 and references therein), where:


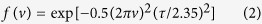


is the so-called form factor (here given for a Gaussian-shaped bunch of FWHM - duration τ) and quantifies the degree of superradiance. It is a dimensionless, frequency-dependent value that, in a simplified view, describes the fraction of the electron bunch that fulfils the superradiance condition. It is directly related to the electron bunch duration τ as illustrated in Fig. 2, where calculated form factors for Gaussian-shaped bunches with an assumed duration of 30 fs, 300 fs and 3000 fs are shown. A 30 fs (FWHM) Gaussian-shaped bunch results in practically fully superradiant emission from the complete electron bunch up to frequencies of 3 THz. For a 300 fs bunch, substantial emission at 0.3 THz and 1 THz can still be expected. In contrast, emission from a 3 ps bunch is already smaller by 2 orders of magnitude even at frequencies as low as 0.3 THz. Due to the quadratic dependency on the bunch charge, emission from the superradiant fraction of the bunch dominates even for small form factors. Assuming a 100 pC bunch charge, corresponding to ***N***_***electron***_ ~ 6 × 10^8^ electrons, even tiny form factors of 10^−3^ already lead to superradiant emission that overtakes the linear *“spontaneous”* emission at the corresponding THz frequency by 5 orders of magnitude.

**Figure 3:**
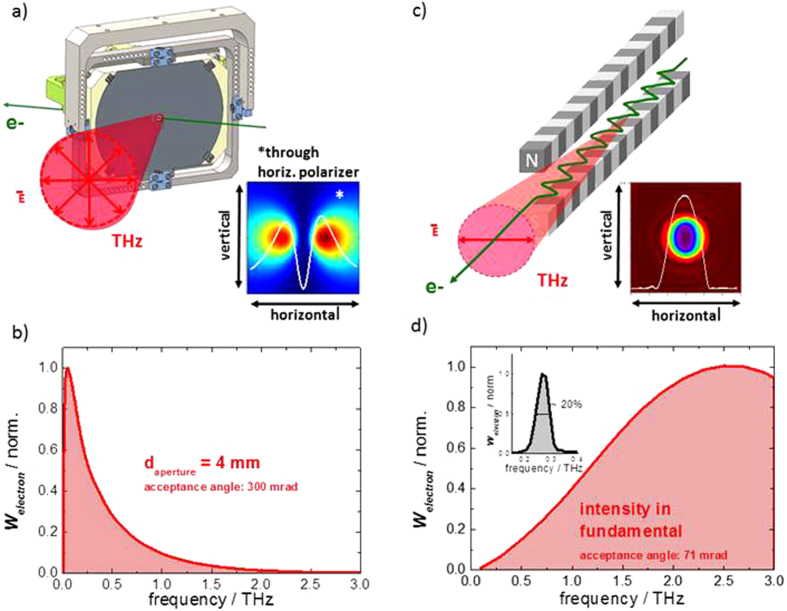
The two THz sources and their calculated emission characteristics. (**a**) Working principle of a diffraction radiator (DR) and a simulated beam profile for the horizontal polarization component. (**b**) Calculation of the intensity spectrum from a DR assuming the screen dimensions and aperture size employed at the prototype facility. (**c**) Working principle of an undulator source and simulation of the beam profile. (**d**) (inset) Calculation of the spectrum of an undulator fundamental for a tune to 0.28 THz. Calculated intensity in the fundamental of undulator tunes between 0.1 and 3 THz (red-shaded). All calculations assume a beam energy of 24 MeV and the corresponding acceptance angles of the DR and the undulator optical beamline.

Presently, two different THz radiation sources are implemented: an 8-period electromagnetic THz undulator^36^ delivering tunable multi-cycle THz pulses, and a diffraction radiator (DR)^37^ providing quasi-single-cycle pulses. Figure 3 shows the working principles and theoretical predictions for the single electron emission characteristics ***W***_***electron***_ of these two radiator types. The reductions due to the finite beam dimensions or the form factor, respectively, are neglected. The first source is based on so-called diffraction radiation which is emitted when a charged particle, or more precisely its Coulomb field passes a boundary between two materials with different dielectric properties. The DR emitter at the prototype facility is an aluminum-coated silicon wafer with a central aperture of 4 mm (see Fig. 3a and supplementary information). The emitted intensity spectrum for a zero-length bunch depends on the size of the DR emitter and the radius of the central aperture through which the electrons pass in combination with the acceptance angle. It can be calculated analytically from an adapted version of the Ginzburg-Frank equation (see^37^ and the supplementary information for details). A calculation for the DR emitter employed at the prototype facility is shown in Fig. 3b. The emitted intensity at low frequencies is governed by the finite outer screen dimensions. It reaches a maximum value around 0.1 THz. Towards higher frequencies the intensity drop is governed by the radius of the central aperture in the DR screen (for details see supplementary information). The aperture size of 4 mm employed at the prototype facility results in a cut-off frequency of about 0.3 THz.

The second source, the undulator, is a classical radiator of synchrotron radiation^38^. It consists of a sequential arrangement of pairs of anti-parallel magnetic dipoles, called undulator periods. During the passage through this magnetic structure electrons experience a Lorentz force of alternating sign and are guided onto a sinusoidal trajectory. Interference between radiation emitted in the forward direction at each turn of the electron trajectory produces a laser-like collimated beam of relatively narrow bandwidth around a fundamental frequency (see Fig. 3c). The frequency can be conveniently tuned via the strength of the magnetic field ***B*** by adjusting the current in the electromagnetic undulator. The magnitude, shape and harmonic content of the spectrum depends on ***B***, the undulator period, the beam energy and the acceptance angle and can be calculated numerically as well as analytically (see^39^,^40^ and supplementary information for details).

**Figure 4:**
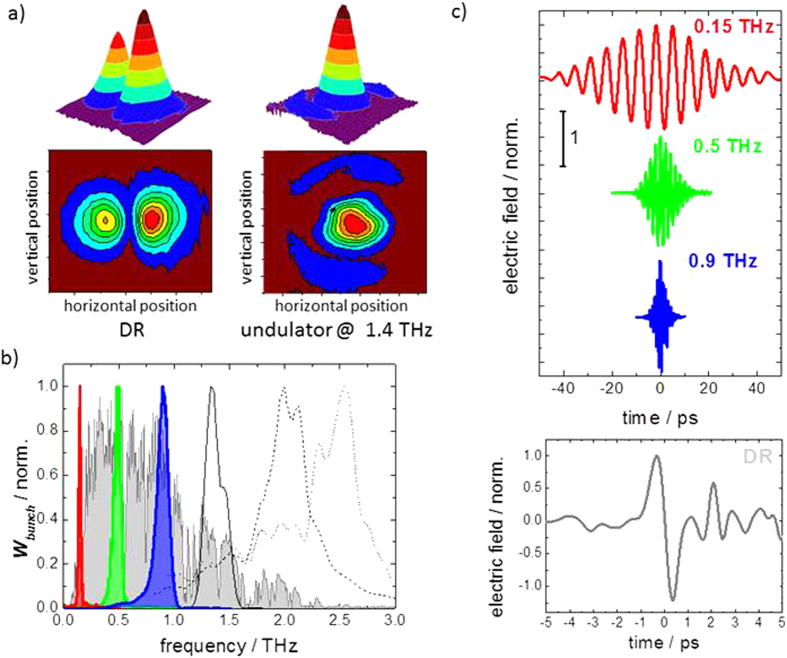
Experimentally observed source properties. (**a**) Beam profiles of DR and undulator radiation determined with a pyroelectric camera. (**b**) Normalized Spectra of a typical broadband DR pulse (grey-shaded) and narrow-band fundamentals of selected undulator tunes (0.15 THz–red-shaded, 0.5 THz–green-shaded, 0.9 THz–blue-shaded, 1.4 THz–black solid, 2.1 THz–dashed, 2.5 THz–dotted). The measurements for tunes to 0.15 THz and 0.5 THz were performed through appropriate band pass filters to remove the higher harmonic content. (**c**) (top) Electric-field transient of undulator pulse for tunes to 0.15 THz, 0.5 and 0.9 THz and (bottom) Electric field transient of a DR pulse taken under ambient conditions. Note that the notches in the frequency domain spectra as well as the ringing after the DR pulse in the time-domain measurements are due to water absorption lines from passage through air. Additional fringes are due to reflections in the ZnTe crystal.

A calculation for the frequency dependence of the intensity within the fundamental for different tunes and typical parameters of the prototype facility is shown in Fig. 3d (red shaded). For a beam energy of 24 MeV the intensity in the fundamental increases with frequency towards a peak value at 2.5 THz. This behavior can be qualitatively understood from undulator theory (see supplementary information for details). Figure 4 shows the experimentally observed properties of diffraction radiation and undulator radiation at the prototype facility.

Beam profiles for both source types have been taken with a pyroelectric camera and are shown in Fig. 4a. In the case of diffraction radiation, the expected two lobes are observed that represent the linear horizontal polarization component filtered out partially by 2 polarizing Brewster angle windows in the optical beamline used to separate the accelerator vacuum from the experimental end stations. The undulator beam profile exhibits the expected bell shaped intensity distribution^39^. Figure 4b shows normalized spectra from the DR source (grey shaded) and a selection of different undulator fundamentals. The observed DR spectrum in Fig. 4b differs significantly from the single-electron emission characteristic shown in Fig. 3b in particular at low THz frequencies. Diffraction effects in the optical transportline, at the sources and at the electro-optic crystal furthermore lead to a sharp low-frequency cut-off at ~0.3 THz. The form factor leads to a reduction of the emitted intensity at high THz frequencies above 1 THz. In contrast, due to the narrow bandwidth the influence of form factor and diffraction effects are less obvious in the normalized undulator spectra. The undulator fundamentals exhibit a bandwidth that is roughly constant around 20% for all frequencies and in good agreement with the calculated single-electron emission characteristics in Fig. 3d. Transient electric fields are shown in Fig. 4c for pulses with undulator tunes to 0.15, 0.5 and 0.9 THz (top) and a typical pulse from the DR radiator (bottom). The expected electric field transients, quasi-single electric-field cycle for the DR and few multicycles for the undulator pulses, are observed.

**Figure 5:**
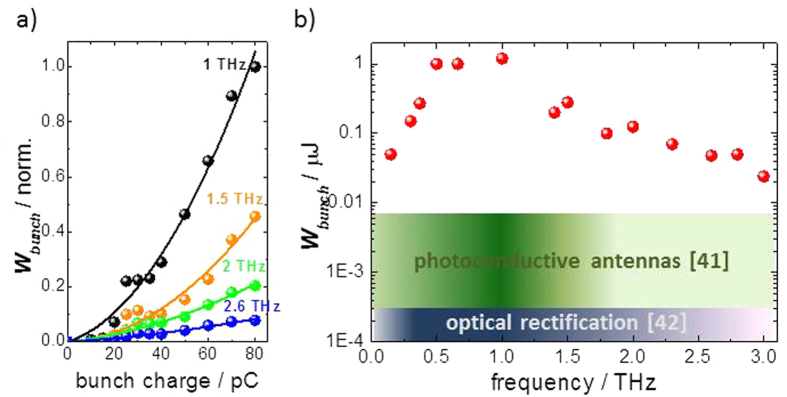
Dependence of pulse energy on bunch charge and THz frequency. (**a**) Measured charge dependence of the emitted THz pulse energy in the fundamental for undulator tunes between 1 and 2.6 THz and 2^nd^ order polynomial fits. (**b**) Maximum pulse energies observed during the first commissioning shifts of the prototype facility (full circles) at a repetition rate of 100 kHz and with a bunch charge of 100 pC. The already demonstrated pulse energies exceed the currently most intense high-repetition-rate laser-based sources^41^,^42^ (shaded) by up to 2 orders of magnitude. Note, the laser-based sources are broadband and have a distribution of spectral weight over many frequencies as indicated by the color tone in the respective shaded areas. Experiments aiming at driving a narrowband low frequency excitation resonantly thereby benefit additionally from the considerably higher provided spectral density.

The individual undulator fundamental harmonics in Fig. 4b are normalized for clarity. The actual pulse energy within these different fundamentals is crucially dependent on the superradiant amplification and is scaling down with frequency and charge. This is demonstrated in Fig. 5a where the charge dependence of the pulse energy is shown for different THz undulator frequencies. The emitted pulse energies scale quadratically with the charge of the electron bunches for all of these frequencies proving the superradiant nature of the observed radiation. However, the actual form factor, or in other words the superradiantly contributing fraction of the bunch at this compression scheme, is reduced substantially between 1 and 2.6 THz, resulting in a drop of the pulse energy by about one order of magnitude. The currently achieved maximum pulse energies from the superradiant undulator are plotted in Fig. 5b. Values of several hundred nJ up to 1 μJ have been achieved routinely, thereby exceeding previous record pulse energies from photoconductive switches (Beck and coworkers, who achieved 6 nJ @ 250 kHz^41^) or from optical rectification of fiber laser pulses (Hoffmann and coworkers, who achieved 0.25 nJ @ 1 MHz^42^) by more than 2 orders of magnitude. The record pulse energy of 1.3 μJ for a bunch charge of 100 pC is achieved at a center frequency of 1 THz. The loss of form factor leads to a relatively sharp decrease of pulse energies above 1 THz in contrast to the single-electron calculation shown in Fig. 3d, while the drop in pulse energy towards lower frequencies corresponds reasonably to the single-electron emission characteristics. Note, that in Fig. 5b energies of narrowband undulator pulses are compared to broad-band table-top sources. Accordingly, the spectral density at an individual fundamental frequency delivered from the undulator is increased (depending on the frequency) by another 1 to 2 orders of magnitude which is of particular advantage for driving resonant processes. Also note that operation at higher charges of up to 1 nC e.g. with the currently commissioned SRF photoinjector^35^, will result in pulse energies in the 100 μJ regime. Also note that recently operation of a DC gun-based photoinjector has been demonstrated that allows generation of bunches with charges up to 2 nC and excellent properties at high-repetition rates, which if employed at TELBE, would immediately result in pulse energies of up to 400 μJ^43^.

**Figure 6:**
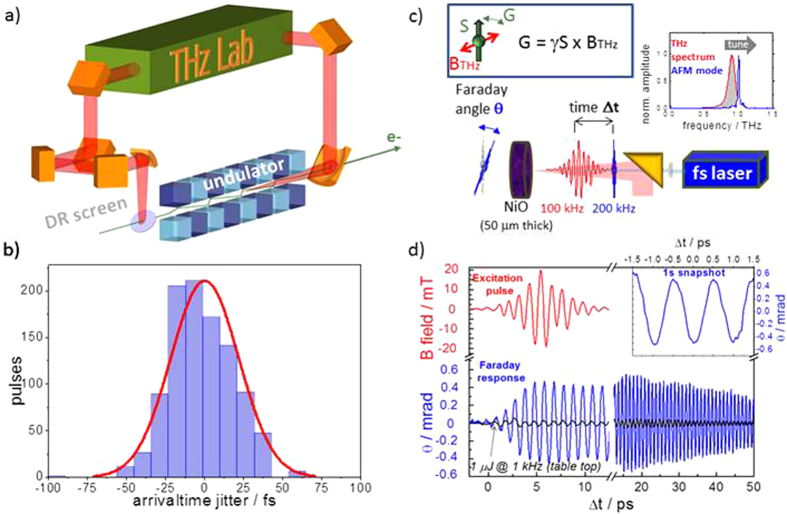
Synchronization and Coherent THz Control benchmark experiment. (**a**) Sketch of the arrangement of THz sources in the accelerator. Pulses emitted from the same electron bunch arrive in the laboratory at the same time. (**b**) Jitter measurement establishing intrinsic synchronization in the few 10 fs regime between undulator and DR pulses. (**c**) Benchmark experiment: The transient B_THz_-field from a multi-cycle THz pulse is utilized to launch a coherent antiferromagnetic spinwave. The spin deflection is probed by the transient Faraday rotation of a timed fs laser. The undulator is tuned in resonance with the AFM mode of NiO at 1 THz and provides a spectral density per pulse that is by a factor 36 larger than achievable from broad-band laser-based THz sources. (**d**) Transient Faraday rotation angle θ (blue-solid) plotted over delay time Δt between THz (red-solid) and laser pulses. The measurement shows that the spin precession evolves coherently over several tens of picoseconds. Due to the orders of magnitude higher spectral intensity at the resonance frequency, the amplitude of the spin deflection is considerably increased compared to coherent excitation by a state-of-the-art high-field table-top THz source of similar pulse energy (black-solid line and^10^). (inset) Snapshots of the spinwave over few cycles taken in less than 1 s. Due to the two orders of magnitude higher repetition rate, measurements can be performed either orders of magnitudes faster or with much higher sensitivity.

In the pump-probe schemes typically employed in experiments utilizing high-field THz pulses, the synchronization of pump and probe pulses is a crucial parameter. One important class of such experiments utilizes multi-cycle THz pulses to target one particular low-energy excitation and probes the initiated dynamics with broad band single cycle THz transients^8^,^15^,^24^. Making use of the fact that radiation pulses emitted from the same electron bunch can be intrinsically synchronized in the few fs regime^44^, such a scheme is realized at the prototype facility using the undulator pulses as pump and the DR pulses as probe (see Fig. 6a). Both sources can be operated in parallel without any observable degradation of their performance. The currently experimentally observed intrinsic jitter is in the few 10 fs regime (see Fig. 6b) and hence slightly larger than expected. We attribute this partially to instabilities of the electron beam position on the few 10 micrometer scale. There is furthermore clear evidence that the jitter is slightly overestimated by the employed technologically challenging 2 pulse single-shot electro-optic sampling measurement (see supplementary information for details).

Another even more crucial parameter for the envisioned experiments aiming at probing coherent control of matter by duty-cycle hungry laser-based techniques is the achievable time-resolution in experiments that involve external fs-laser systems as a probe. In order to benchmark the performance of the prototype source we have performed a coherent control experiment and compared the measurement with the results of experiments performed at 1 kHz utilizing a state-of-the-art table top source based on optical rectification of an optical femtosecond laser pulse with tilted pulse front in LiNbO_3_ 8. In the benchmark experiment the transient magnetic field of a carrier-envelope-phase-stable narrowband THz undulator pulse at a repetition rate of 100 kHz tuned in resonance with the antiferromagnetic mode (AFM) of NiO was used to coherently drive a spin excitation with a resonance frequency close to 1 THz (see Fig. 6c). The time-dependent transient magnetic field B in the THz pulse exerts a Zeeman torque on to the electron spin which results in a collective spin precession. The maximum pulse energy employed in the experiment was 1 μJ which was focused onto a spot diameter of 1 mm on the sample surface. Inside the NiO crystal the incident pulse results in a maximum transient magnetic field of up to 39 mT. The dynamics was probed by monitoring the transient Faraday effect utilizing a synchronized NIR fs laser at a repetition rate of 200 kHz.

The experimentally observed transient Faraday rotation angle is shown in Fig. 6d. Two immediate observations are made. Firstly, the higher spectral density at 1 THz results in a significantly increased Faraday signal and, therefore, THz-induced spin deflection angle as compared to the experiment with the table-top THz source albeit of an equivalent pulse energy. Secondly, the much higher repetition rate allows us to either probe the dynamics much faster as demonstrated by the snapshot of the spinwave shown in the inset of Fig. 6d or to achieve a significantly improved signal-to-noise ratio. Ultimately of higher importance is that the TELBE facility can be combined with duty-cycle-hungry specific spectroscopic techniques operating at repetition rates in the few 100 kHz to few 10 MHz regime, such as time resolved photoelectron spectroscopy. In the specific case of THz control of spin excitations laser-based TR Brillouin or TR Raman scattering^25^ would e.g. allow the direct observation of the dephasing via magnon-phonon scattering processes. The achievable time-resolution in a THz pump laser probe experiment was verified to be better than 15 fs (rms) (see supplementary information for details).

To summarize, we have demonstrated for the first time parallel operation of two superradiant high-field THz sources at a compact quasi-cw electron accelerator at unprecedented high repetition rates and benchmarked the performance by exercising a real-world ultra-fast coherent THz control experiment. The prototype facility provides a world-wide unique combination of up to 100 kV/cm fields at up to MHz repetition rates. The provided THz fields can be significantly further upscaled when operating with higher bunch charges (see Fig. 1b). Intrinsically synchronized single- and multi-cycle high-field THz pulses can be generated at user-determined repetition rates. The fundamental working principle allows operation with different radiator types. This opens up the opportunity to provide a large variety of different THz pulse properties at one time from one electron beam ranging from extreme narrow bandwidth sources (utilizing corrugated waveguides^45^), over few-multi cycle (undulators^46^) to quasi-single cycle pulses (TR/DR sources^32^,^33^, bending magnets^47^ or edge radiators^48^) and from linear to radial and circular polarization. With an overall length of less than 20 meters such superradiant, quasi-cw THz facilities can be compact, cost- and space-efficient^49^. It is important to note that the driving compact SRF technology is being continuously further developed for various upcoming X-ray FEL projects^50^,^51^. The performance of SRF accelerators will hence be dramatically improved, while their cost will be further reduced in the coming years. The prototype facility presented in this article is highly complementary to recently developed laser-based high-field THz sources operational at lower repetition rate^52^,^53^,^54^. Its considerably higher duty cycle opens the opportunity to employ much more specific spectroscopy probes for the THz driven dynamics. The combination of selective THz pump with specific duty-cycle hungry probe techniques will pave the way for gaining deeper fundamental insights into the many fascinating THz driven phenomena which so far can only be described superficially^8^,^9^,^22^,^23^. Furthermore, operated as multi-user facilities this type of sources will be accessible also to non-laser experts and hence will widen the scope of science with high-field THz pulses.

## Methods

The experiments were performed at the prototype Terahertz facility at ELBE (TELBE) recently installed at the quasi-cw linear SRF electron accelerator ELBE^34^. The THz pulses from both sources were transported into a dedicated laser laboratory by transport lines based on reflective optics. In each beam path a set of 2 Brewster windows from z-cut crystalline quartz separate the accelerator vacuum from the experimental set-ups and supress the vertical polarization component. All THz photon-diagnostics presented in this article have been performed in the laboratory. Measurements have been performed in a nitrogen atmosphere or in vacuum to reduce water absorption where possible. Spectral properties have been determined making use of one commercial FTIR spectrometer (Bruker 80 V) and several purpose-built electro-optic sampling set-ups primarily utilizing 100 fs long 800 nm laser pulses from a Coherent Reg A laser-amplifier-system. The laser system was locked to the accelerator via a stabilized optical fibre link^55^ or via a commercial RF Lock. Power/pulse energy measurements have been performed using a commercial sensor^56^ that has been specifically calibrated for the experiments by the Physikalisch-Technische Bundesanstalt^57^. At and above 1 THz frequency the measurements have an accuracy of 1.7% while the uncertainty is larger at lower frequencies^56^,^58^. The pulse energies presented in the article are as measured in the laboratory and are not corrected for losses by the transport into the laboratory. Timing and time-domain measurements where performed utilizing single-shot and sequential electro-optic sampling schemes. Beam profiles have been measured with a commercial pyroelectric camera.

## Additional Information

**How to cite this article**: Green, B. *et al.* High-Field High-Repetition-Rate Sources for the Coherent THz Control of Matter. *Sci. Rep.* **6**, 22256; doi: 10.1038/srep22256 (2016).

## Supplementary Material

Supplementary Information

## References

[b1] Zhang X. C. *et al.* Generation and femtosecond electromagnetic pulses from semiconductor surfaces. Appl. Phys. Lett. 56, 1011 (1990).

[b2] Wu Q. & Zhang X.-C.. Free-Space Electro-Optic Sampling of Terahertz Beam. Appl. Phys. Lett. 67, 3523 (1995).

[b3] Chan W. L., Deibel J. & Mittleman D. M.. Imaging with terahertz radiation. Reports on progress in physics 70, 1325 (2007).

[b4] van Exter M. & Grischkowsky D.. Optical and electronic properties of doped silicon from 0.1 to 2 THz. Appl. Phys. Lett. 56, 1694 (1990).

[b5] Schmuttenmaer C. A.. Exploring dynamics in the far-infrared with terahertz spectroscopy. Chem. Rev. 104, 1759 (2004). 15080711 10.1021/cr020685g

[b6] Yu B. L. *et al.* Torsional Vibrational Modes of Tryptophan Studied by Terahertz Time-Domain Spectroscopy. Biophys. Journ. 86, 1649 (2004). 10.1016/S0006-3495(04)74233-2 PMC1304000 14990492

[b7] Yu B. L. *et al.* Terahertz absorption spectrum of D2O vapor. Opt. Comm. 258, 256 (2006).

[b8] Kampfrath T., Nelson K. A. & Tanaka K.. Resonant and nonresonant control over matter and light by intense terahertz transients. Nature Photon. 7, 680 (2013).

[b9] Hwang H. Y. *et al.* A review of non-linear terahertz spectroscopy with ultrashort tabletop-laser pulses. Journ. Mod. Opt, doi: 10.1080/09500340.2014.918200 (2014).

[b10] Kampfrath T. *et al.* Coherent THz control of an antiferromagnetic spinwave. Nat. Phot. 5, 34 (2011).

[b11] Rini M. *et al.* Control of the electronic phase of a manganite by mode-selective vibrational excitation. Nature 449, 72 (2007). 17805291 10.1038/nature06119

[b12] Fausti D. *et al.* Light-Induced Superconductivity in a Stripe-Ordered Cuprate. Science 331, 189 (2011). 21233381 10.1126/science.1197294

[b13] Liu M. *et al.* Terahertz-field-induced insulator-to-metal transition in vanadium dioxide metamaterial. Nature 487, 345 (2012). 22801506 10.1038/nature11231

[b14] Pashkin A. *et al.* Electric and magnetic terahertz nonlinearities resolved on the sub-cycle scale. New. J. Phys. 15, 065003 (2013).

[b15] Kaiser S. *et al.* Optical properties of a Vibrationally Modulated Solid State Mott Insulator. Scientific Reports 4, 3823 (2014). 24448171 10.1038/srep03823 PMC3898202

[b16] Kaiser S. *et al.* Optically induced coherent transport far above Tc in underdoped YBa_2_Cu_3_O_6+δ_. Phys. Rev. B 89, 184516 (2014).

[b17] Hu W. *et al.* Optically enhanced coherent transport in YBa_2_Cu_3_O_6.5_ by ultrafast redistribution of interlayer coupling. Nat. Mat. 13, 705 (2014). 10.1038/nmat3963 24813422

[b18] Kim K.-T. *et al.* High-power femtosecond-terahertz pulse induces a wound response in mouse skin. Scientific Reports 3, 2296 (2013). 23907528 10.1038/srep02296 PMC3731731

[b19] Smallwood C. L. *et al.* Tracking Cooper Pairs in a Cuprate Superconductor by Ultrafast Angle-Resolved Photoemission. Science 336, 1137 (2012). 22654053 10.1126/science.1217423

[b20] Wang Y. & Zhang F.-C.. Momentum-resolved electronic relaxation dynamics in d-wave supercondutcors. Phys. Rev. B. 89, 094519 (2014).

[b21] Yang L. X. *et al.* Ultrafast Modulation of the Chemical Potential in BaFe_2_As_2_ by Coherent Phonons. Phys. Rev. Lett. 112, 207001 (2014).

[b22] Orenstein J.. Ultra-fast spectroscopy of quantum materials. Physics Today 65, 44 (2012).

[b23] Armitage N. P.. Dynamic stabilization. Nat. Mat. 13, 665 (2014). 10.1038/nmat3995 24813419

[b24] Dienst A. *et al.* Optical excitation of Josephson plasma solitons in a cuprate superconductor. Nat. Mat. 12, 535 (2013). 10.1038/nmat3580 23524373

[b25] Saichu R. P. *et al.* Two-Component Dynamics of the Order Parameter of High Temperature Bi_2_Sr_2_CaCu_2_O_8+δ_ Superconductors Revealed by Time-Resolved Raman Scattering. Phys. Rev. Lett. 102, 177004 (2009). 19518817 10.1103/PhysRevLett.102.177004

[b26] Eisele M. *et al.* Ultrafast multi-terahertz nano-spectroscopy with sub-cycle temporal resolution. Nat. Photon. 8, 841 (2014).

[b27] Schmitt F. *et al.* Transient Electronic Structure and Melting of a Charge Density Wave in TbTe_3_. Science 19, 1649 (2008). 18703710 10.1126/science.1160778

[b28] Hashimoto M. *et al.* Energy gaps in high-transition-temperature cuprate superconductors. Nat. Phys. 10, 483 (2014).

[b29] Kottke T. *et al.* Thinner, Smaller, Faster: IR Techniques To Probe the Functionality of Biological and Biomimetic Systems. Ang. Chemie Int. Ed. 49, 5416 (2010). 10.1002/anie.200907114 20818765

[b30] Heyne K. *et al.* Real-Time Tracking of Phytochrome’s Orientational Changes During Pr Photoisomerization. Journ. Am. Chem. Soc. 134, 1408 (2012). 10.1021/ja209413d 22229806

[b31] Williams G. P.. High power synchrotron radiation sources. Phil. Trans. R. Soc. Lond. A 362, 403 (2004). 10.1098/rsta.2003.1325 15306529

[b32] Shen Y. *et al.* Nonlinear Cross-Phase Modulation with Intense Single-Cycle Terahertz Pulses. Phys. Rev. Lett. 99, 043901 (2007). 17678365 10.1103/PhysRevLett.99.043901

[b33] Wu Z. *et al.* Intense terahertz pulses from SLAC electron beams using coherent transition radiation. Rev. Sci. Instr. 84, 022701 (2013). 10.1063/1.4790427 23464183

[b34] Gabriel F. *et al.* The Rossendorf Radiation Source ELBE and its FEL projects. Nucl. Instrum. Methods Phys.Res. B 161, 1143 (2000).

[b35] Arnold A. *et al.* Overview on superconducting photoinjectors. Phys. Rev. ST Accel. Beams 14, 024801 (2011).

[b36] Ostenfeld C. W. O. & Pedersen M.. 300 mm Electromagnetic Wiggler for ELBE, Proceedings of FEL13, New York 24.8. –31.8.2013, TUPSO55 (2013).

[b37] Casabuoni S. *et al.* Far-Infrared Transition and Diffraction Radiation Part I: Production, Diffraction Effects and Optical Propagation, TESLA Report 2005–15, DESY (Date of access: 28/01/2016): http://flash.desy.de/sites2009/site_vuvfel/content/e403/e1644/e1173/e1174/infoboxContent1352/tesla2005-15.pdf (2005).

[b38] Clark J. A.. The Science and Technology of Undulators and Wigglers. Oxford University Press 584, New York (2004).

[b39] Asgekar V. *et al.* Interference effects in super-radiant THz sources. Infrared Phys. Technol. 64, 26 (2014).

[b40] Geloni G. *et al.* A method for ultra-short pulse-shape measurements using far infrared coherent radiation from an undulator. Nucl.Instr. Meth. B 528, 184 (2004).

[b41] Beck M. *et al.* Impulsive terahertz radiation with high electric fields from an amplifier-driven large-area photoconductive antenna. Optics Express 18, 9251 (2010). 20588772 10.1364/OE.18.009251

[b42] Hoffmann M. C. *et al.* Fiber laser pumped high average power single-cycle terahertz pulse source. Appl. Phys. Lett. 93, 141107 (2008).

[b43] Gulliford C. *et al.* Demonstration of cathode emittance dominated high bunch charge beams in a DC gunbased photoinjector. App. Phys. Lett. 106, 094101 (2015).

[b44] Fruehling U. *et al.* Single-shot THz-driven X-ray streak camera. Nat. Photon. 3, 523 (2009).

[b45] Bane K. L. F. & Stupakov G.. Terahertz radiation from a pipe with small corrugations. Nucl. Instrum. Meth. A 667, 67 (2012).

[b46] Gensch M. *et al.* New infrared undulator beamline at FLASH. Infrared Phys. Technol. 51, 423 (2008).

[b47] Carr G. L. *et al.* High-power terahertz radiation from relativistic electrons. Nature 420, 153 (2002). 12432385 10.1038/nature01175

[b48] Tavella F. *et al.* Few Femtosecond Timing at 4^th^ Generation X-ray Lightsources. Nat. Photon. 6, 162 (2011).

[b49] Nasse M. J. *et al.* FLUTE: a versatile linac-based THz source. Rev. Sci. Instr. 84, 022705 (2013). 10.1063/1.4790431 23464187

[b50] Altarelli M. *et al.* The European X-ray free-electron laser facility in Hamburg. Nucl. Instrum. Meth. B 269, 2845 (2011).

[b51] Reich S.. Labs vie for X-ray source. Nature 500, 13 (2013). 23903728 10.1038/500013a

[b52] Kunitski M. *et al.* Optimization of single-cycle terahertz generation in LiNbO_3_ for sub-50 femtosecond pump pulses. Optics Express 21, 6826 (2013). 23546064 10.1364/OE.21.006826

[b53] Huang S. W. *et al.* High conversion efficiency, high energy terahertz pulses by optical rectification in cryogenically cooled lithium niobate. Opt. Lett. 38, 796 (2013). 23455302 10.1364/OL.38.000796

[b54] Vicario C. *et al.* GV/m Single-Cycle Terahertz Fields from a Laser-Driven Large-Size Partitioned Organic Crystal. Phys. Rev. Lett. 111, 213901 (2014).

[b55] Loehl F. *et al.* Electron Bunch Timing with Femtosecond Precision in a Superconducting Free-Electron Laser. Phys. Rev. Lett. 104, 144801 (2010). 20481941 10.1103/PhysRevLett.104.144801

[b56] OPHIR Photonics. High Sensitivity Thermal Sensors, 33 (Date of access: 28/01/2016): http://www.ophiropt.com/laser/pdf/3A_3A-P_3A-P-THz_3A-FS_3A-P-FS-12.pdf (2015).

[b57] Steiger A. *et al.* Traceable THz power measurement from 1 THz to 5 THz. Opt. Expr. 21, 14466 (2013). 10.1364/OE.21.014466 23787634

[b58] Müller R. *et al.* Novel detectors for traceable THz power measurements. J. Infrared. Milli. TeraHz Waves 35, 659 (2014).

