# Supplementary material for: High-Field High-Repetition-Rate Sources for the Coherent THz Control of Matter
Source: Sci Rep. 2016 Feb 29;6:22256. doi: 10.1038/srep22256 (PMC4770290; doi:10.1038/srep22256)
Supplement: Supplementary Information [file srep22256-s1.pdf]

Supplementary Information:

High-Field High-Repetition-Rate Sources for the Coherent THz Control of Matter

B. Green[1+], S. Kovalev[1*], V. Asgekar[2,14+], G. Geloni[3], U. Lehnert[1], T. Golz[2], M. Kuntzsch[1,15], C. Bauer[4], J. Hauser[1], J. Voigtlaender[1], B. Wustmann[1], I. Koesterke[1], M. Schwarz[5], M. Freitag[1], A. Arnold[1], J. Teichert[1], M. Justus[1], W. Seidel[1], C. Ilgner[1], N. Awari[1,16], D. Nicoletti[6], S. Kaiser[6,17,18], Y. Laplace[6], S. Rajasekaran[6], L. Zhang[6], S. Winnerl[1], H. Schneider[1], G. Schay[7], I. Lorincz[7], A.A. Rauscher[7], I. Radu[8], S. Mährlein[9], T.H. Kim[10], J.S. Lee[10], T. Kampfrath[9], S. Wall[11], J. Heberle[4], A. Malnasi-Csizmadia[7], A. Steiger[12], A.S. Müller[5], M. Helm[1,15], U. Schramm[1,15], T. Cowan[1,15], P. Michel[1], A. Cavalleri[6], A.S. Fisher[14], N. Stojanovic[2], M. Gensch[1]*

1 Helmholtz-Zentrum Dresden-Rossendorf, Bautzner Landstr. 400, 01328 Dresden, Germany
2 Deutsches Elektronen Synchrotron, Notkestr. 85, 22607 Hamburg, Germany
3 European X-FEL GmbH, Albert-Einstein-Ring 19, 22761 Hamburg, Germany
4 Freie Universität Berlin, Arnimallee 14, 14195 Berlin, Germany
5 Karlsruhe Institute of Technology, Kaiser Str.12, 76131 Karlsruhe, Germany
6 Max Planck Institute for the Structure and Dynamics of Matter, Luruper Chaussee 149, 22761 Hamburg, Germany
7 Eotvos Lorand University, Pazmany s 1/C. 1117 Budapest, Hungary
8 Helmholtz-Zentrum Berlin, Albert-Einstein Str.15, 12489 Berlin, Germany
9 Fritz-Haber-Institut der Max-Planck-Gesellschaft, Faradayweg 4-6, 14195 Berlin, Germany
10 Gwangju Institute of Science and Technology (GIST), Gwangju 500-712, Korea
11 ICFO – Institute of Photonic Sciences, Av. Carl Friedrich Gauss 3, 08860 Castelldefels (Barcelona), Spain
12 Physikalisch-Technische Bundesanstalt (PTB), Abbestr 2-12, 10587 Berlin, Germany
13 SLAC National Accelerator Laboratory, 2575 Sand Hill Rd, Menlo Park, California 94025, USA
14 S. P. Pune University, Pune 411 007, India
15 Technische Universität Dresden, 01062 Dresden, Germany
16 University of Groningen, 9747 AG Groningen, Netherlands
17 Max Planck Institut für Festkörperforschung, Heisenbergstr. 1, 70569 Stuttgart, Germany
18 Universität Stuttgart, Pfaffenwaldring 57, 70550 Stuttgart, Germany

*corresponding authors
+contributed equally to this work

***email: s.kovalev@hzdr.de and m.gensch@hzdr.de**

1. Definitions of intensity used in the article

In this paper, for reasons of simplicity, measured pulse energies and calculated quantities are compared by making use of normalization and the term "W_X / norm." However, in the present paragraph, for reasons of completeness, we would like to detail the actual quantity that has been calculated.

Experimentally derived values are either determined from spectrally resolved time or frequency domain measurements (fig. 4b and fig. S5b) or are determined with calibrated power/pulse energy meters (fig. 5a,b).

Concerning the numerical calculation of the undulator emission characteristics in Fig 3d & Fig S4a, and Fig S5b, the physical quantity actually calculated was expressed in terms of the photon flux, N_{ph}/s/0.1%BW/mm^2. In the case of the red curve in fig. 3d and both curves in fig. S5a, we plot the area under the first harmonic as a function of the frequency.

Also, in case of the analytical calculation of the diffraction radiation in fig. 3b and fig. S2 the physical quantity is N_{ph}/s/0.1%BW/mm^2.

2. Emission characteristics of the Diffraction Radiator

Diffraction Radiation (DR) is emitted when the Coulomb field of a relativistic particle passes the boundary between two materials with different dielectric properties. Its spectral content and angular distribution can be calculated analytically by adapting the classical Ginsburg-Frank formula [S1,37] to include finite screen dimensions and a central aperture. Then the number of photons collected in area S and frequency band $\Delta\nu_{THz}$ is:

$$W_{DR}(\nu_{THz}) = \frac{f_{rep}}{\hbar}\frac{\Delta\nu_{THz}}{\nu_{THz}}\frac{e^2}{\epsilon_0 c 4\pi^3}\int\frac{(\beta\sin\theta)^2}{(1-\beta^2\cos^2\theta)^2}\left[T\left(\frac{2\pi\nu_{THz}b}{c},\theta\right)-T\left(\frac{2\pi\nu_{THz}a}{c},\theta\right)\right]^2 R^{-2}dS$$

$$T\left(\frac{2\pi\nu_{THz}x}{c},\theta\right) = \frac{2\pi\nu_{THz}x}{c\beta\gamma}J_0\left(\frac{2\pi\nu_{THz}x\sin\theta}{c}\right)K_1\left(\frac{2\pi\nu_{THz}x}{c\beta\gamma}\right) + \frac{2\pi\nu_{THz}x}{c\beta^2\gamma^2\sin\theta}J_1\left(\frac{2\pi\nu_{THz}x\sin\theta}{c}\right)K_0\left(\frac{2\pi\nu_{THz}x}{c\beta\gamma}\right)$$

where ν_{THz} is the THz frequency, *e* is the elementary charge, *c* is the speed of light, ε_0 dielectric constant of vacuum ,γ is the Lorentz factor, $\beta = v_e/c$, θ is the angle from the center of the source to a point on the detector, *a* is the screen radius, *b* is the aperture radius, R is the distance to the detector, *dS* is the surface area of the detector and f_{rep} is the repetition rate of the electron accelerator. *J* and *K* are Bessel functions.

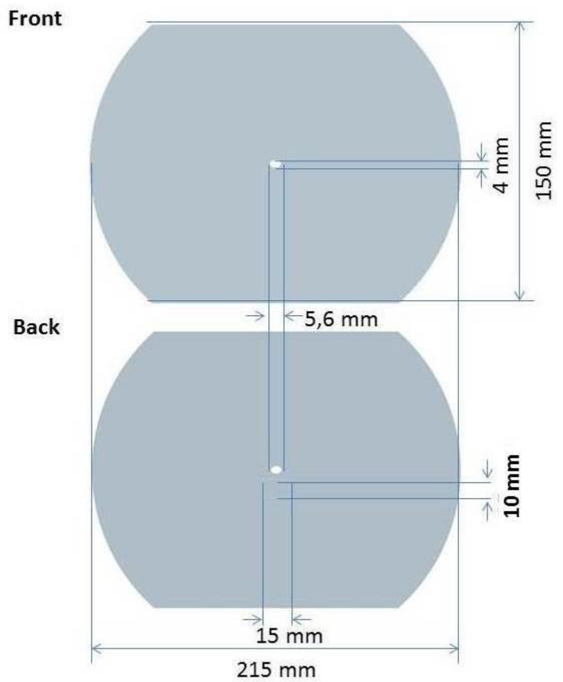

Figure S1: sketch of the DR screen in the prototype facility. The screen consists of a 734 µm thick silicon substrate coated with 200 nm of aluminum. It is mounted at 45 degrees to the incoming electron beam so that its projects to a circle when viewed from this direction. Note that, beside a central aperture of a projected diameter of 4 mm, there also exists an area of 10 x 15 mm where the silicon substrate is thinned down to 100 µm thickness for measurements in transition radiation (TR) geometry.

Utilizing this formula the emission characteristic can be calculated and has been plotted in figure 3b of the article. The outer dimensions of the screen in the

prototype facility shown in figure S1 lead to a low frequency cut-off below 0.1 THz.
Note, the spectral content of diffraction radiation at high THz frequencies is restricted by the aperture diameter (see figure S2a). Assuming emission from a single electron, a transition radiator (case *d=0*) is a *white emitter* since no frequency dependence is observed above a cut-off of 0.1 THz caused by the finite screen diameter. Introducing into this ideal case an aperture of diameter d= 4 mm (used in the prototype facility) results in a high frequency cut-off (50%) at ~ 0.3 THz. This can be understood from the equation above and from a rule of thumb [S2] based on the distance between the moving charge and the screen, which predicts no significant emission for frequencies $\nu > \gamma c/\pi d$.

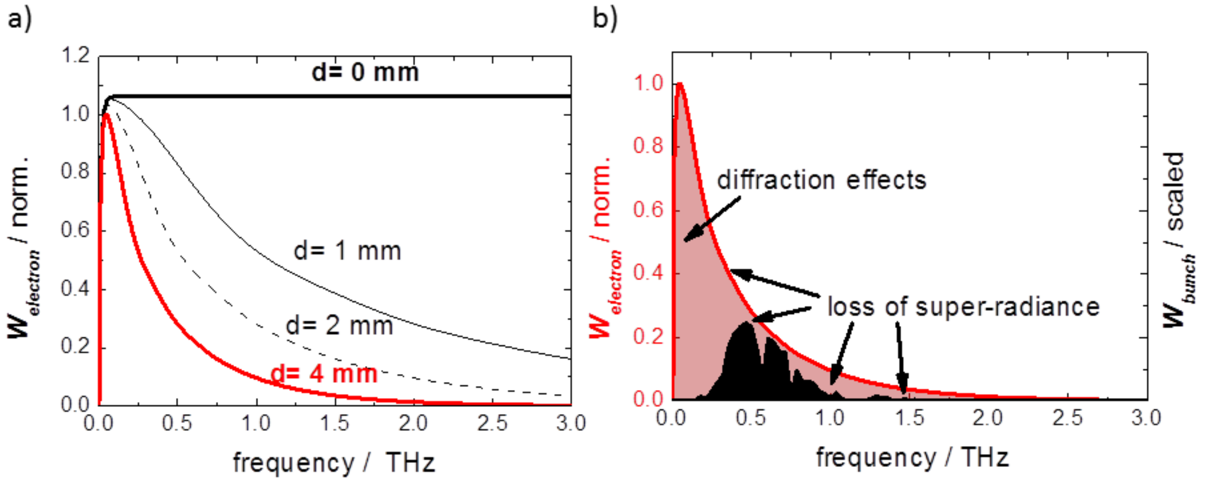

Figure S2: a) Influence of the aperture size on the emission characteristics assuming a 24 MeV electron beam and the DR screen dimensions at the prototype facility. b) Comparison of the calculated spectrum (red-shaded) and a scaled typical measurement (black-shaded). The calculations assume a zero-length bunch, an acceptance angle of 300 mrad and neglect diffraction in the optical transport. The notches in the measured spectra are due to water absorption lines.

As can be seen in figure S2b the measured spectral shape of the DR radiation differs significantly from the calculation. At low frequencies the spectrum is predominantly affected by diffraction losses during the optical transport to and at the detector. The differences at high frequencies are primarily caused due to the loss of super-radiance. Note that in the case of DR both, the transverse and longitudinal electron bunch sizes affect the superradiance.

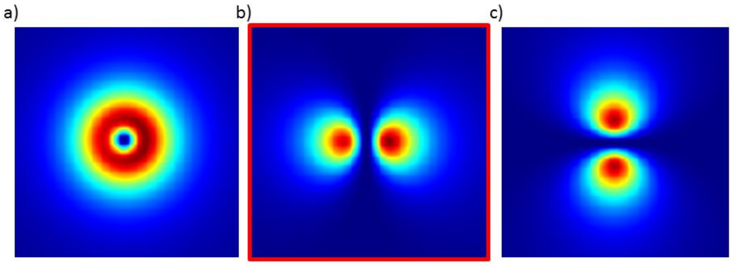

Figure S3: Beamprofiles for diffraction radiation for a) the full beam, b) the horizontal polarization component and c) the vertical polarization component.

As shown in figure 3a, diffraction radiation exhibits radial polarization with a characteristic donut-like shaped beam profile with a slight asymmetry due to the 45 degree tilt of the screen (see figure S3a). If predominantly the horizontal polarization component is transported, as is the case at the prototype facility, the beam profile transforms into one with 2 horizontal lobes (see figure S3b). Passing only the vertical polarization component leads to 2 vertical lobes as shown in figure S3c.

3. Emission characteristics of superradiant undulator radiation

Undulators are the most popular source types of synchrotron radiation at 3rd generation light sources and also are at the heart of free electron lasers. Accordingly a wealth of literature describes the concept and their use in great detail, although focusing typically on their application to the generation of X-ray radiation. The use of undulators for the generation of super-radiant THz radiation is a relatively recent utilization [46] and in contrast not yet dealt with in the text books or the review articles. Here we focus on the special features of the undulator developed for the prototype facility.

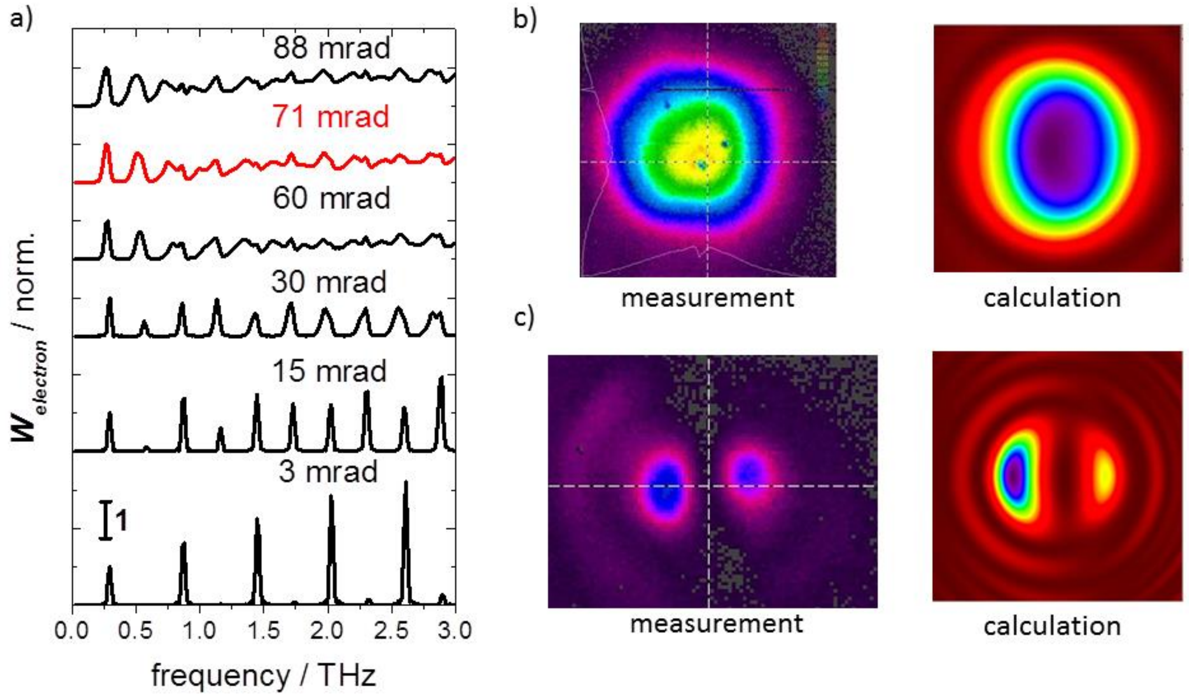

Figure S4: a) Calculated harmonic spectra of the undulator radiation for different acceptance angles. The acceptance angle realized in the prototype facility is 71 mrad (marked in red). b) Calculation and measurement of a beamprofile of the fundamental for a tune to 0.37 THz. c) Calculation and measurement of a beamprofile of the 2nd harmonic of a tune to 0.68 THz. All measurements were performed at beam energy of 24 MeV and through a band pass filter to remove the harmonic contents of the spectrum. All calculations assumed a beam energy of 24 MeV and neglect superradiant effects.

Undulator radiation is emitted by relativistic electrons forced onto a sinusoidal path by a periodic arrangement of anti-parallel magnetic dipoles (see figure 3c). The emitted intensity consists of a narrow-band spectrum around a fundamental frequency and of higher harmonics [38]. The frequency of the fundamental is given by the following formula:

$$\nu = \frac{c}{\lambda_w} \frac{2\gamma^2}{\left(1 + \frac{1}{2}K^2\right)} \qquad \text{(S1)}$$

where γ *is the Lorentz-factor*, λ_w is the undulator period length. K is the undulator parameter and defined as:

$$K = eB_w\lambda_w/(2\pi m_e c) = 0.934\ B_w(T)\ \lambda_w(cm) \qquad \text{(S2)}$$

where B_w is the amplitude of the undulator magnetic field.

The frequency of the fundamental is tunable by either the electron beam energy or the magnetic field of the dipoles (as can be deduced from equation S1). At the prototype facility an

electromagnetic undulator is used [36] and by changing the magnet current, *K* and hence ν can be adjusted over a wide frequency range between 0.1 and 5 THz. The undulator consists of 8 undulator periods, where each period consists of a pair of anti-parallel magnetic dipoles. Additional half periods at both ends and magnetic correction coils are used to match the electron beam before and after the undulator to the beamline axis and to correct minor imperfections of the magnetic field distribution (for details see [36]). Important for the emission characteristic are the comparatively small number of periods (X-ray undulators or undulator modules in FELs typically have 100 periods or more) and the large acceptance angle of the quasi-optical transport of 71 mrad. For a given beam energy the undulator spectrum can be calculated numerically (see [39] and references therein) with high accuracy. One such calculation is shown in figure S4a presenting the harmonic spectrum for different acceptance angles. As can be seen, the harmonic spectrum is strongly dependent on the acceptance angle and exhibits both even and odd harmonics at larger acceptance angles, while towards small angles only odd harmonics at reduced intensity are emitted. In figure S4b measured and calculated beamprofiles for the fundamental and the second harmonic are shown. The second harmonic (and in fact all even harmonics) exhibit a minimum intensity in the center. In contrast, the fundamental (and all odd harmonics) have a bell-shape beamprofile with the peak intensity in the center. As explained in Section 1 of the Supplementary information, in Fig S4a we plot the normalized photon flux as a function of the frequency. As the acceptance angle is increased, more even-harmonic photons are going to be collected. This explains the relative increase of the even harmonic peaks. Note that the odd harmonics tend to decrease as we go e.g. from 3mrad to 15mrad. This can be ascribed to the fact that we plot our normalized data per unit surface, and is due to the fact that the acceptance increases well beyond the main radiation (central) cone.

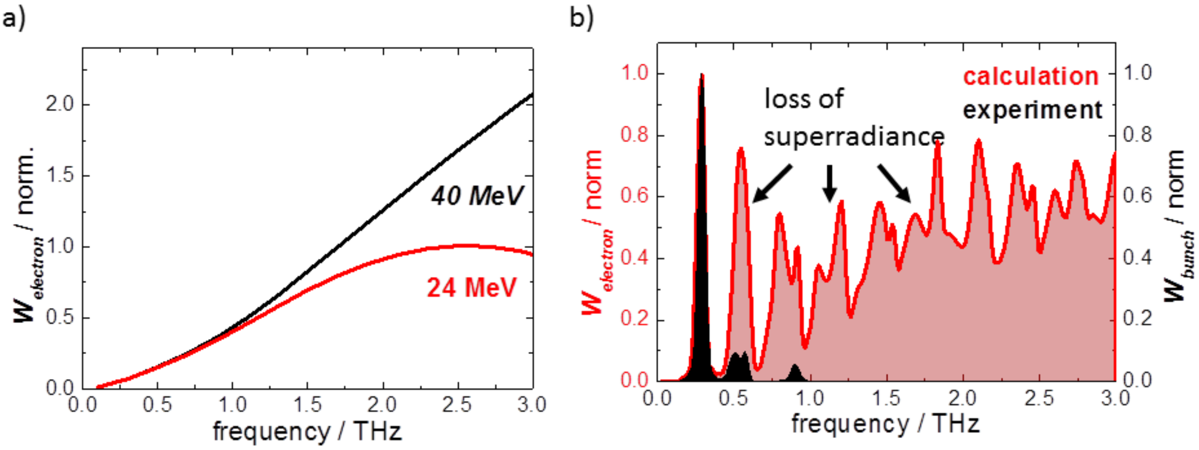

Figure S5: a) Scaling of the pulse energy in the undulator fundamental with the beam energy. Shown are calculations for 24 MeV and 40 MeV. b) Comparison of a calculated spectrum (red-shaded) and a measurement for a tune to 0.28 THz (black-shaded). Note all the calculations were performed with an acceptance angle of 71 mrad.

Conceptually important is that the calculated emission at higher frequencies is relatively strongly dependent on the beam energy as is shown in figure S5a. When aiming for optimal emission at higher THz frequencies the maximum available beam energy should be used (40 MeV at the prototype facility). The general frequency dependence of the pulse energy within a fundamental shown in figure 3d and S5a can be understood as follows. The figures show the photon flux through the systems acceptance angle integrated in frequency around the first harmonic as a function of the fundamental tune i.e. the area under the first harmonic in Fig 3d (black curve - inset) for different fundamental tunes. Increasing the energy of the fundamental, the frequency range in the fundamental increases, while the angular size of the central cone decreases. This leads to an overall increase in the value of the integral. However, while the energy of the fundamental increases, the value of the undulator parameter *K* decreases below unity, which tends to decrease the flux. As a result the characteristic curve reaches a maximum and then decreases. Since the frequency where

the maximum is reached depends on *K*, it is shifted to higher frequencies with increasing electron beam energy. In figure S5b a comparison of the calculated spectrum and a measurement is shown. The harmonic spectrum is shortened considerably as the finite electron bunch duration reduces superradiance at higher THz frequencies.
Note that, in contrast to the diffraction radiator, the size of the electron beam does not affect the undulator emission characteristics. The influence of the finite transverse size of the electron beam on the intensity (here we neglect the divergence and the energy spread of the electron beam) depends on its ratio to the radiation diffraction size. Neglecting the interference from other sources, as done everywhere in this article, the undulator radiation diffraction size at the resonance wavelength λ is ~ sqrt(L_w*λ/2π), where L_w is the length of the undulator. For the shortest wavelength of 100 μm (corresponding to a frequency of 3 THz), the diffraction size is 6 mm. Since the electron beam diameter is about 1 mm, its effect on the coherent radiation intensity can be safely neglected. Note that the undulator used here is made up of 8 periods. This number, albeit relatively small, has the effect of increasing the diffraction size of the radiation and justifies the filament-beam approximation.

4. Parallel operation of DR and undulator source

One important aspect of our proposed concept is the opportunity to operate several THz radiators in parallel. In an earlier paper [39] we have discussed that under certain circumstances such an arrangement can lead to interference effects between the different sources. In addition, particularly at low electron beam energies, the emission of substantial THz pulse energies at one emitter may lead to a modification of the electron bunch properties and hence influence the super-radiant emission of subsequent sources. In the case of the prototype facility we have the compact arrangement shown in figure S6a. The DR screen is situated 2.78 meters upstream of the center of the undulator and can be moved in and out the electron beams. This feature has been utilized to study experimentally the impact of the DR screen on the undulator performance is. In general, given that the electron beam was properly steered through the 4 mm aperture in the screen we observed no influence of the DR screen on the performance of the undulator neither in its spectral properties nor pulse energy/intensity. This is confirmed from even more sensitive time-domain measurements where we could not observe evidence of residual THz pulses beyond the few percent regime (see figure S6b).

Since the DR screen is indeed emitting half of its intensity into forward direction collinear to the electron beam, this finding is initially surprising. In the time domain at an electron beam energy of 24 MeV, one would expect to observe this collinear pulse roughly 1 ps before the undulator pulse. Indeed we find evidence of a single cycle pulses preceding the undulator pulse by roughly 1 ps (see fig. S6b). The amplitude of the electric field is almost 2 orders of magnitude smaller than that of the peak-field in the undulator pulse, explaining the negligible influence of the DR screen on the undulator performance. We attribute this low intensity to severe losses of intensity of the coherent diffraction radiation in the electron and photon beamlines. In particular the undulator photon beamline is matched for the divergence angle and source point of the undulator radiation and does not favor transport of diffraction radiation from the DR screen or from the out coupling mirror.

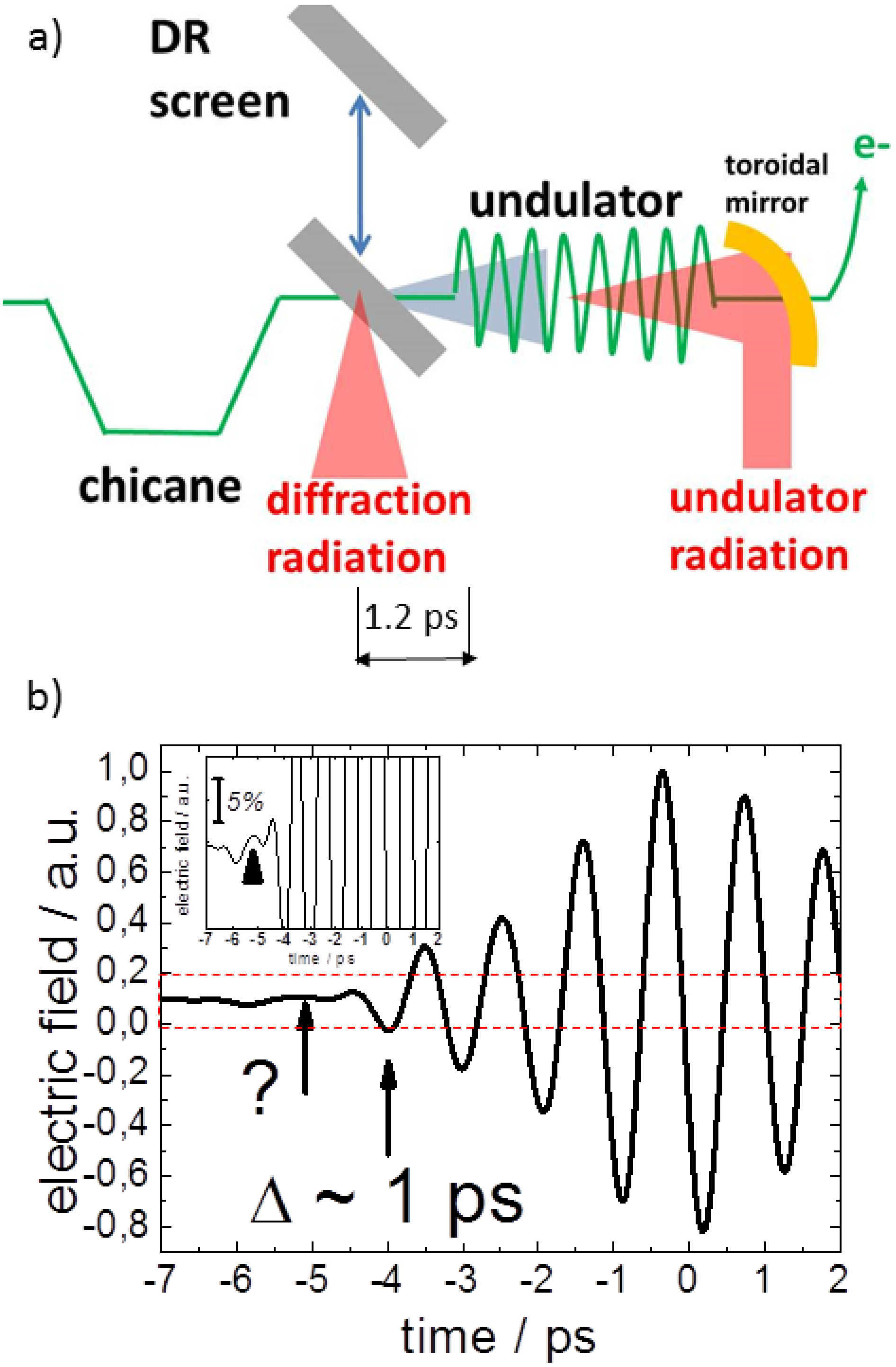

Figure S6: a) Sketch of the sequential arrangement of DR screen and undulator in the ELBE accelerator. By retracting the DR screen from the beam its effect on the undulator performance can be determined. Note that forward CDR radiated by the DR screen collinearly with the electron beam which should arrive roughly 1 ps earlier for a beam energy of 24 MeV (shaded in blue). b) Time-domain analysis of the time immediately prior to an undulator pulse when tuned to 1 THz. A small prepulse is observed at roughly the expected time for forward CDR.

5. Time resolution in THz pump laser probe experiments

The achievable time resolution in pump probe experiments utilizing the THz pulses generated from the accelerator and the ultrashort pulses from external laser systems is a crucial parameter. At TELBE we achieve a time resolution of better than 15 fs (rms) by pulse to pulse arrivaltime measurements based on the spectral-decoding [S3] and quasi-online data analysis. The, by this scheme, achievable time resolution in a THz undulator pump laser probe experiment has been benchmarked by performing a series of sequential electro-optic sampling measurement on the undulator THz pulses. In the measurements (see schematic in figure S7a) the single cycle pulse from the DR radiator acts as time-prompt to clock the arrivaltime of the undulator pulse to the NIR probe laser exactly like in the pilot coherent control experiment shown in figure 6. We utilize the intrinsic few 10 fs synchronization of DR and undulator pulses when emitted from the same electron bunch [44]. We then deliberately shifted the CDR pulse/timeprompt in few fs steps by an optomechanical delay against the NIR probe laser and perform electro-optic sampling measurements of the undulator pulse at each delay step. The shift of the undulator against the NIR probe laser is then taken as a measure for the achieved synchronization and is plotted in figure S7b.

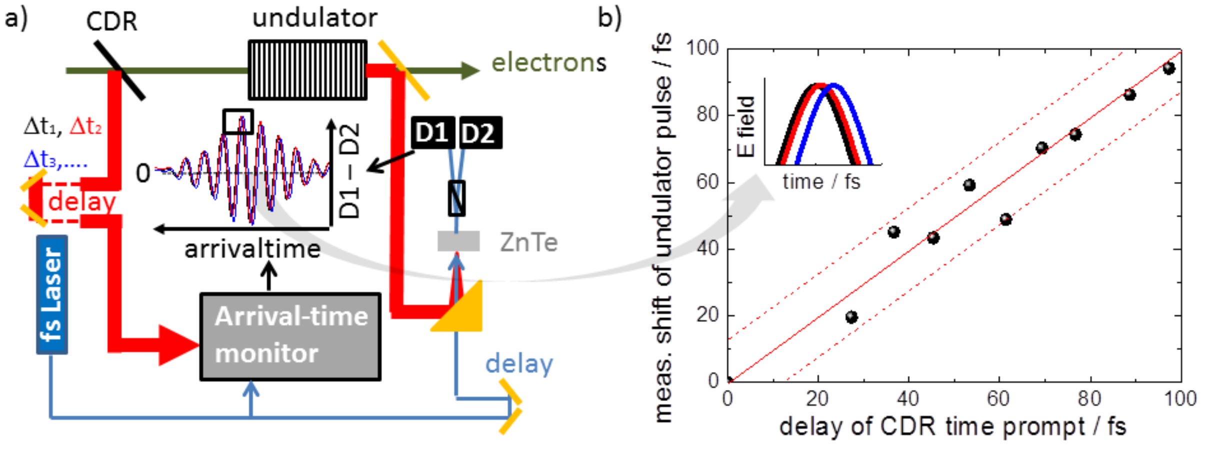

Figure S7: a) Sketch of the scheme employed for the determination in THz pump laser probe experiments and b) measured time shift of the undulator pulse against the probe laser upon delaying the CDR time prompt. Establishing an uncertainty/jitter of less than 35 fs (peak-to-peak) or 15 fs (rms).

Note the achieved time resolution of 35 fs (peak-to-peak)/15 fs (rms) includes the small but nonzero intrinsic jitter between DR and undulator pulses. In fact the experiment shown in figure S7 proves that the larger value of 25 fs (rms) derived from the technologically more challenging 2 pulse single-shot spectral decoding measurement shown in figure 6b is an overestimate. In future experiments we plan to perform the arrivaltime measurement utilizing the actual pump pulse and thereby hope to achieve a time resolution even in the few fs regime.

[S1] I.M. Frank, V.I. Ginzburg, *Radiation of a uniform moving electron due to its transition from one medium into another*, *J. Phys. (USSR)*, **9** (1945), 353.

[S2] A.P. Potylitsyn, *Transition radiation and diffraction radiation. Similarities and differences*, Nucl. Instrum. Meth. B **145** (1998), 169.

[S3] Z. Jiang, X. C. Zhang, *Measurement of Spatio-Temporal Terahertz Field Distribution by Using Chirped Pulse Technology*, IEEE Journal of Quantum Electronics, **36** (2000), 1214.
